# Supplementary figures and images for: Today’s computing challenges: opportunities for computer hardware design
Source: PeerJ Comput Sci. 2021 Mar 30;7:e420. doi: 10.7717/peerj-cs.420 (PMC8022507; doi:10.7717/peerj-cs.420)

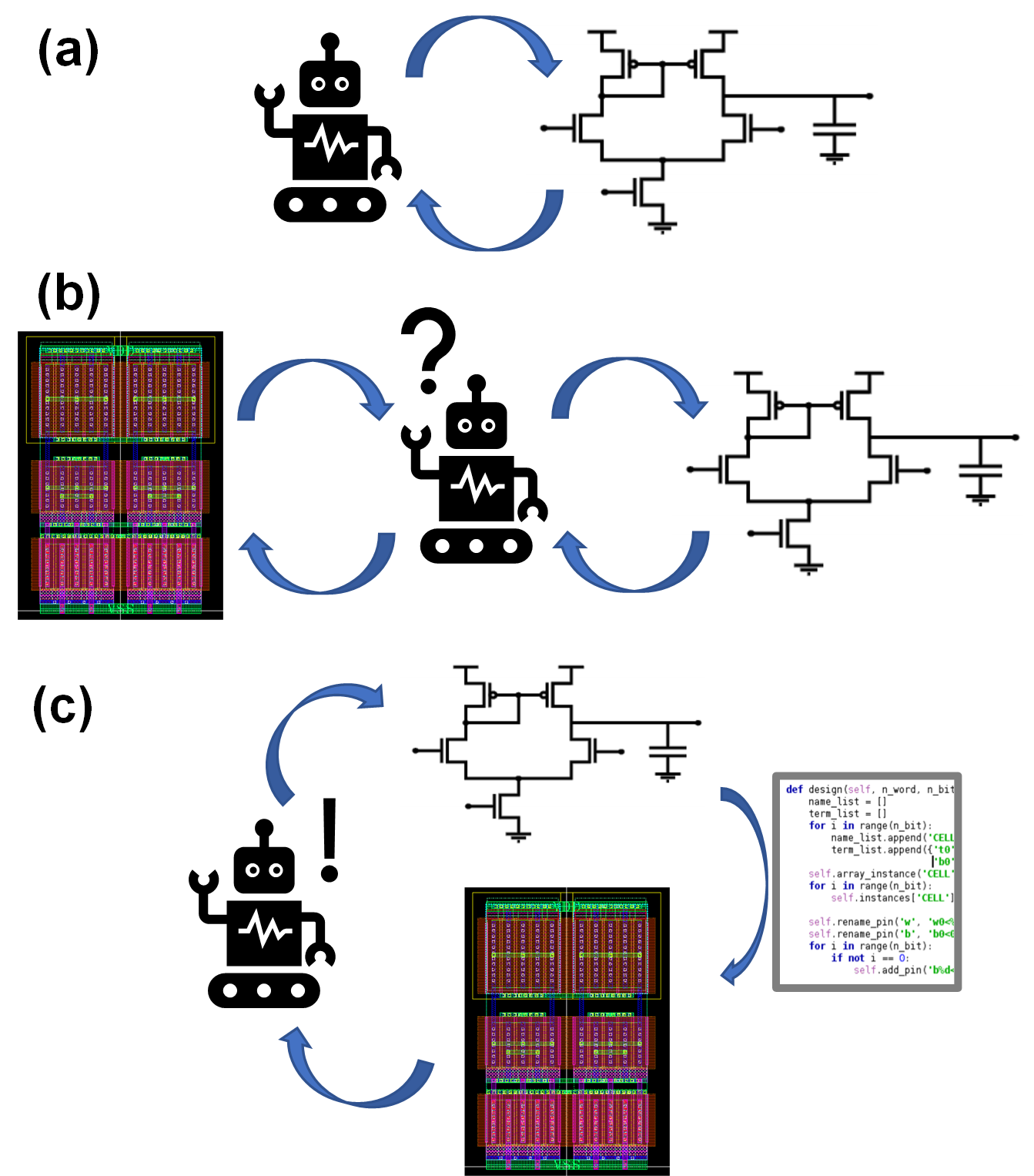

Supplement: Supplemental Information 1 [file peerj-cs-07-420-s001.png]

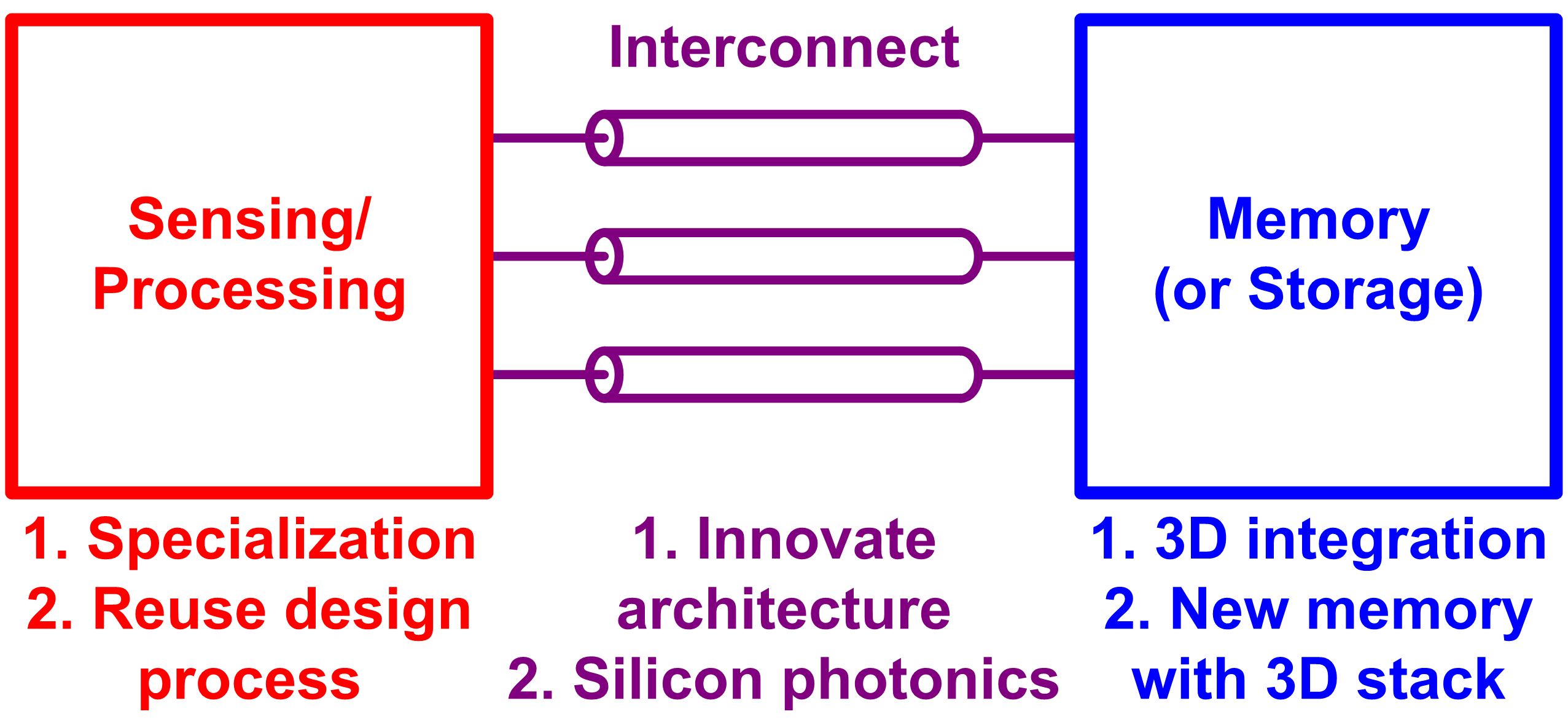

Supplement: Supplemental Information 2 [file peerj-cs-07-420-s002.png]
